# Can IgG4 Levels Identify the Ulcerative Colitis Subtype of Inflammatory Bowel Disease?

**DOI:** 10.14740/gr648w

**Published:** 2015-04-03

**Authors:** Ricardo Jacaranda Faria, Cintia Mendes Clemente, Fabiana P. Carneiro, Leopoldo Santos-Neto

**Affiliations:** a Universidade de Brasilia, Brasilia, Distrito Federal, Brazil; b Hospital Universitario de Brasilia, Brasilia, Distrito Federal, Brazil; c Hospital de Base do Distrito Federal, Brasilia, Distrito Federal, Brazil

**Keywords:** Inflammatory bowel disease, IgG4, Autoimmune pancreatitis

## Abstract

**Background:**

Pancreatitis and exocrine pancreatic insufficiency may occur as extraintestinal manifestations of inflammatory bowel disease. Recently, autoimmune pancreatitis and colitis have been described as presentations of IgG4-related disease. IgG4+ plasma cells have been identified in colon tissue from patients with refractory forms of inflammatory bowel disease. The presence of elevated serum/tissue levels of IgG4 and the frequency of exocrine pancreatic insufficiency in inflammatory bowel disease are still a source of controversy. Our aim was to investigate the meaning of elevated IgG4 levels in patients with inflammatory bowel disease.

**Methods:**

A cross-sectional study analyzed 56 patients with a diagnosis of inflammatory bowel disease recruited by convenience sampling from two tertiary centers in Midwestern Brazil. All patients underwent fecal pancreatic elastase testing for detection of exocrine pancreatic insufficiency and serum IgG4 measurement. Findings were correlated with clinical and epidemiological data and disease activity.

**Results:**

Elevated serum IgG4 levels were found in 10 patients, and were most frequent in ulcerative colitis (nine cases), with a prevalence ratio of 16.42 (95% CI: 3.32 - 79.58). Ten patients (10 of 56, 17.8%) were diagnosed with exocrine pancreatic insufficiency, which did not correlate with disease activity, and serum IgG4 levels.

**Conclusion:**

Exocrine pancreatic insufficiency is prevalent in patients with inflammatory bowel disease, but it is not associated with elevated serum IgG4 levels. The high prevalence of elevated serum IgG4 in ulcerative colitis suggests that this parameter has potential for use as a diagnostic biomarker.

## Introduction

The prevalence of exocrine pancreatic insufficiency in patients with inflammatory bowel disease ranges from 8% to 50% [1, 2]. Diagnosis is challenging, as exocrine pancreatic insufficiency may be mistaken for diarrhea due to decompensated inflammatory bowel disease or simply for treatment-refractory inflammatory bowel disease [1, 3, 4].

Exocrine pancreatic insufficiency in patients with inflammatory bowel disease is multifactorial, and its etiology includes adverse effects of drugs such as azathioprine and mesalazine, biliary disease, duodenal involvement in Crohn’s disease [5], and IgG4-related disease (IgG4-RD) [6, 7].

One of the major manifestations of IgG4-RD [8] is autoimmune pancreatitis [9]. These patients are at a 15-fold greater risk of developing inflammatory bowel disease as compared with the general population [6]. It is unclear whether measurement of IgG4+ plasma cells in serum and tissue samples could play an important role in the etiological workup of the pancreatitis and consequent exocrine pancreatic insufficiency often found in patients with inflammatory bowel disease. Whether the presence of elevated serum IgG4 levels and colonic infiltration by IgG4+ plasma cells might characterize a new phenotype of inflammatory bowel disease, IgG4-related colitis, is still controversial.

The objective of this study was to describe the clinical and epidemiological profile of patients with inflammatory bowel disease and exocrine pancreatic insufficiency and ascertain whether exocrine pancreatic insufficiency is associated with the diagnosis of IgG4-RD.

## Methods

The study sample comprised patients recruited from two referral centers for inflammatory bowel disease care in Brasilia, Brazil, Hospital Universitario de Brasilia (affiliated with Universidade de Brasilia) and Hospital de Base (affiliated with the Brazilian Federal District Department of Health), from January 2010 through December 2011.

All patients met diagnostic criteria for Crohn’s disease or ulcerative colitis [10]. The criteria for exclusion were age less than 18 years, pregnancy, or alcoholism, the latter defined as a CAGE score of > 1 [11]. The Montreal criteria were used to determine the extent of Crohn’s disease and classify its phenotype [12].

Disease activity was measured by means of the Crohn’s disease activity index in patients with Crohn’s disease and with the Lichtiger clinical activity index [13] in patients with ulcerative colitis. All patient interviews were structured and conducted by the same investigator (RJF).

Fecal pancreatic elastase measurement was performed by the ELISA method (BioServ Diagnostics Fecal Elastase-1 ELISA stool test, BioServ Analytics and Medical Devices Ltd, Rostock, Germany), with a normal value > 200 μg/g stool [14]. Watery stool samples were excluded from analysis, as they might produce false-positive elastase measurements.

Serum IgG4 levels were measured by nephelometry (BN II System, Siemens Healthcare Diagnostics Products GmbH, Marburg, Germany), with a normal value of 6.9 - 88 mg/dL. We established a cutoff point of > 140 mg/dL, in accordance with the HISORt criteria [5, 15].

Colon biopsy samples and surgical specimens were assessed for IgG4-positive plasma cell expression by an experienced, blinded pathologist (FP) using immunohistochemical methods. Specimens were incubated with rabbit monoclonal antibody to human IgG4 (100 μL) (Epitomics, USA) and cells counted in three high-power fields, with a cutoff point of ≥ 10 IgG4+ plasma cells per field.

All patients with acute pancreatitis [16] or incidental findings consistent with pancreatic injury on computed tomography (CT) or ultrasonography performed at the time of data collection underwent magnetic resonance cholangiopancreatography.

Data were initially analyzed by descriptive statistics, with calculation of means, medians, and standard deviations of the variables of interest. Inferential analysis consisted of multiple Poisson regression [17], with calculation of prevalence ratios and their corresponding 95% confidence intervals, for each response variable, namely, reduced fecal elastase and elevated serum IgG4.

This study was approved by the Research Ethics Committees of both participating institutions and written informed consent was obtained from all patients.

## Results

A total of 80 patients with inflammatory bowel disease were recruited, 56 of whom completed the study. Overall, 17 were excluded for missing data, four declined to participate, and three died during the data collection period (one due to pulmonary embolism and two of abdominal sepsis).

The study sample was mostly composed of women (63%). Mean (SD) age was 43 (14) years, and only 36.7% of patients with ulcerative colitis and 30.8% of those with Crohn’s disease had active disease (Table 1). Regarding extent of involvement, most patients with Crohn’s disease had predominantly colonic lesions (76%), with non-stricturing, non-penetrating disease (54%) (Table 2). In the ulcerative colitis subsample, most patients had left-sided colitis (60%).

**Table 1:** Clinical and Epidemiologic Profile of Patients With Inflammatory Bowel Disease (n = 56)

Variable	UC^a^ (n = 30)	CD^b^ (n = 26)	Overall (n = 56)
Females	19	16	35
Age (years)^c^	42 (14)	44 (15)	43 (14)
Reduced fecal elastase^d^	5	5	10
Elevated serum IgG4^e^	9	1	10
Active disease^f^	14	8	22
Extraintestinal manifestations	3	0	3
Time elapsed since diagnosis (months)^c^	67 (71)	69 (46)	68 (61)

^a^Ulcerative colitis. ^b^Crohn’s disease. ^c^Mean (standard deviation). ^d^Number of patients with fecal elastase level < 200 μg/g of stool. ^e^Number of patients with serum IgG4 level > 140 mg/dL. ^f^Number of patients with active disease as defined by the Lichtiger clinical-activity index (UC) or the Crohn’s disease activity index (CD).

**Table 2:** Extent (Location and Behavior) of Crohn’s Disease According to the Montreal Classification (n = 26)

Variable	n
Location	
Ileal	6
Colonic	19
Ileocolonic	1
Upper GI^a^ tract	0
Perianal	0
Behavior	
Non-stricturing, non-penetrating	14
Stricturing	6
Penetrating	6

^a^Gastrointestinal.

Regarding extraintestinal manifestations and comorbid autoimmune diseases, only one patient (in the ulcerative colitis group) had primary sclerosing cholangitis. One patient in the Crohn’s disease group had autoimmune hepatitis with overlap syndrome, and one patient with ulcerative colitis had comorbid systemic lupus erythematosus.

All patients had received some form of pharmacotherapy for their inflammatory bowel disease. The most commonly prescribed medications were mesalazine (n = 54), azathioprine (n = 33), prednisone (n = 31), and biologics (n = 13). One patient had received budesonide.

**Figure 1:**
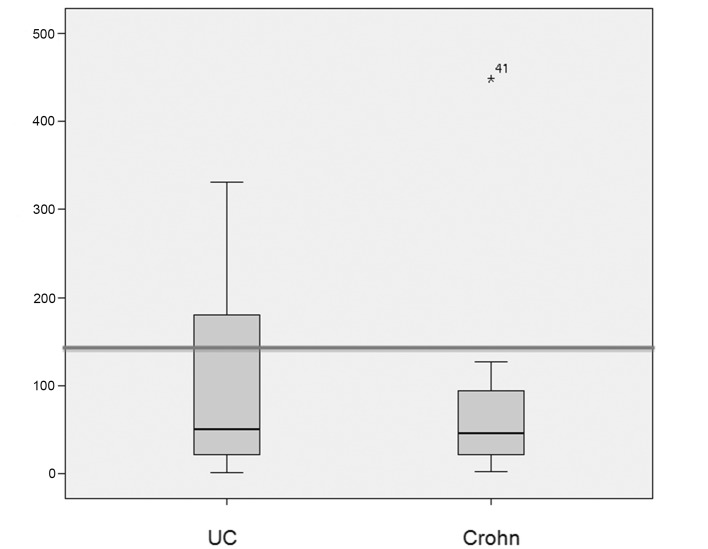
Box plots showing the distribution of IgG4 levels (in mg/dL) in patients with ulcerative colitis (UC) and Crohn’s disease (CD). The median IgG4 level was 50 mg/dL in the UC group and 46 mg/dL in the CD group. IgG4 levels > 140 mg/dL were significantly more prevalent in patients with UC (P = 0.0006). The line represents the 140 mg/dL cutoff.

**Figure 2:**
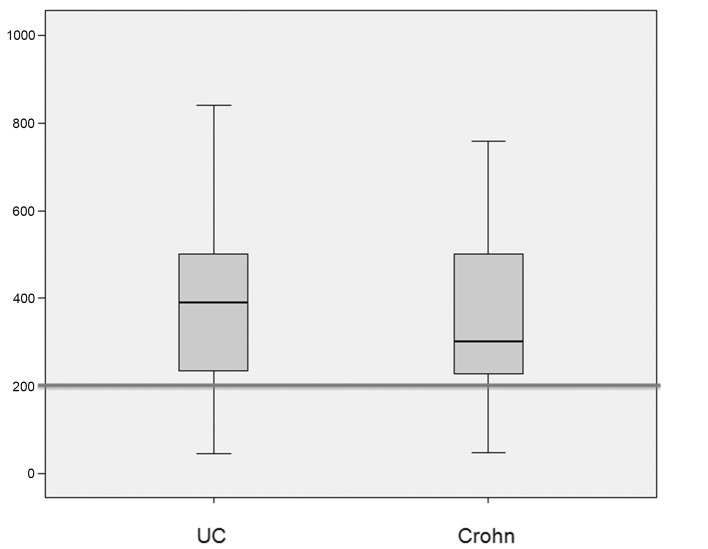
Box plots showing the distribution of fecal pancreatic elastase levels (in μg/g of stool) in patients with ulcerative colitis (UC) and Crohn’s disease (CD). The median elastase level was 391 μg/g in the UC group and 301 μg/g in the CD group. There was no significant between-group difference in the prevalence of levels < 200 μg/g (P = 0.7).

The median (standard deviation) IgG4 levels were 50 ± 99 mg/dL and 46 ± 86 g/dL in patients with ulcerative colitis and Crohn’s disease respectively. The median (standard deviation) elastase levels were 391 ± 183 μg/g in patients with ulcerative colitis and 301 ± 173 μg/g in patients with Crohn’s disease (Fig. 1, 2).

Elevated serum IgG4 levels were detected in 10 patients (nine in the ulcerative colitis group and one in the Crohn’s disease group). Patients with ulcerative colitis had a significantly higher prevalence of IgG4 elevation as compared with the Crohn’s disease group (P = 0.006) (Table 3). Azathioprine use was significantly more common among patients with elevated IgG4 (P = 0.017). On the other hand, disease activity was not significantly associated with serum IgG4 levels (P = 0.8) (Table 4).

**Table 3:** Association Between Sociodemographic and Clinical Variables and Serum IgG4 > 140 mg/dL in Patients With Inflammatory Bowel Disease and Prevalence Ratios After Multiple Poisson Regression

	n (%)	Prevalence, n (%)	95% CI	PR^a^	95% CI^b^	P-value
Sex						
Male	21 (37%)	6 (28.57%)	8.64 - 48.51	1.00		
Female	35 (63%)	4 (11.43%)	0.55 - 22.3	1.89	0.63 - 5.63	0.2000
Disease						
UC^c^	30 (53.5%)	9 (30%)	13.08 - 46.92	16.42	3.32 - 79.58	0.0006
CD^d^	26 (46.4%)	1 (3.85%)	0.00 - 11.47	1.00		
Activity						
Remission	37 (66%)	6 (16.22%)	3.96 - 28.47	1.01	0.31 - 3.35	0.9000
Active	19 (33%)	4 (21.05%)	2.14 - 39.97	1.00		
Elastase						
> 200 μg/g	46 (82.2%)	8 (17.39%)	6.09 - 28.69	1.00		
< 200 μg/g	10 (17.8%)	2 (20.0%)	0.00 - 45.58	0.74	0.18 - 3.02	0.7000
Azathioprine						
No	23 (41%)	3 (13.04%)	0.00 - 27.74	1		
Yes	33 (59%)	7 (21.21%)	6.82 - 35.6	4.8	1.32 - 17.39	0.0017
Corticosteroids						
No	25 (40%)	5 (20%)	3.82 - 36.18	3.86	1.01 - 14.65	0.0470
Yes	31 (60%)	5 (16%)	2.77 - 29.49	1		

^a^Prevalence ratio (multiple Poisson regression). ^b^95% confidence interval, corrected (multiple Poisson regression). ^c^Ulcerative colitis. ^d^Crohn’s disease.

**Table 4:** Association Between Sociodemographic and Clinical Variables and Fecal Elastase < 200 μg/g in Patients With Inflammatory Bowel Disease and Prevalence Ratios After Multiple Poisson Regression

	n (%)	Prevalence, n (%)	95% CI	PR^a^	95% CI^b^	P-value
Sex						
Male	21 (37%)	4 (19%)	1.72 - 36.38	1.26	0.39 - 4.06	0.7000
Female	35 (63%)	6 (17%)	4.26 - 30.02	1.00		
Disease						
UC^c^	30 (53.5%)	5 (16.67%)	2.91 - 30.43	1.00		
CD^d^	26 (46.4%)	5 (19.23%)	3.60 - 34.86	1.25	0.22 - 7.19	0.8000
Activity						
Remission	37 (66%)	7 (18.92%)	5.9 - 31.94	1.16	0.33 - 4.16	0.8000
Active	19 (33%)	3 (15.79%)	0.00 - 32.71	1.00		
IgG4						
> 140 mg/dL	10 (17.8%)	2 (20%)	0.00 - 45.58	1.23	0.21 - 7.15	0.8000
< 140 mg/dL	46 (82.2%)	8 (17.39%)	6.09 - 28.69	1.00		
Azathioprine						
No	23 (41%)	4 (17.39%)	1.41 - 33.37	1		
Yes	33 (59%)	6 (21.21%)	4.60 - 31.76	1.18	0.24 - 5.93	0.8000
Corticosteroids						
No	25 (40%)	5 (20%)	3.82 - 36.18	1.00		
Yes	31 (60%)	5 (16.13%)	2.77 - 29.49	1.04	0.23 - 4.73	0.9000

^a^Prevalence ratio (multiple Poisson regression). ^b^95% confidence interval, corrected (multiple Poisson regression). ^c^Ulcerative colitis. ^d^Crohn’s disease.

Exocrine pancreatic insufficiency was diagnosed in 10 patients. It was secondary to acute pancreatitis in one patient, secondary to chronic obstructive pancreatitis in another, and in the remaining eight, etiology could not be determined. The patient who developed exocrine pancreatic insufficiency after chronic obstructive pancreatitis was ultimately diagnosed with intraductal papillary mucinous neoplasm, underwent pancreaticoduodenectomy, and has been asymptomatic for 8 months on pancreatic enzyme supplementation.

Of the 26 colon tissue samples obtained, 25 were endoscopic biopsy specimens; only one patient yielded a surgical sample (ileocolectomy specimen). Samples from three patients exhibited inflammatory infiltration with ≥ 10 IgG4+ plasma cells per high-power field, but none of these patients had elevated serum IgG4 and only one had exocrine pancreatic insufficiency.

**Figure 3:**
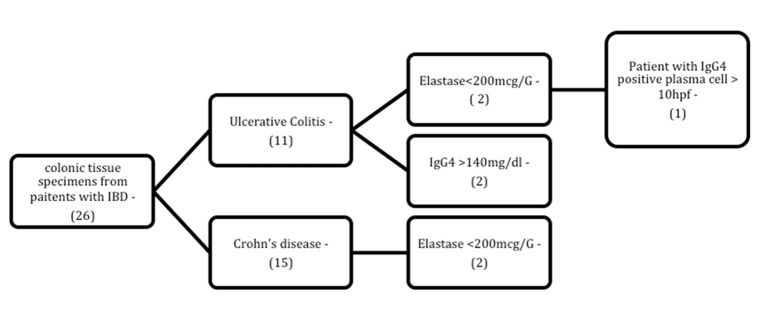
Flowchart of patients with inflammatory bowel disease and abnormal laboratory findings (IgG4/elastase) among those for whom immunohistochemical assessment of tissue IgG4 levels was available. N: number of patients; HPF: high-power field.

Exocrine pancreatic insufficiency was detected in two patients each in the ulcerative colitis and Crohn’s disease groups. Of these four patients, only one (from the ulcerative colitis group) was given a tentative diagnosis of IgG4-RD due to the presence of IgG4+ plasma cell infiltration in tissue samples (Fig. 3); the remaining three had normal findings.

The etiological distribution of disease in the four patients with acute pancreatitis or incidentally detected pancreatic lesions was as follows: one case each of adverse drug reaction, intraductal papillary mucinous neoplasm, IgG4-related autoimmune pancreatitis, and idiopathic disease.

## Discussion

Pancreatic manifestations of inflammatory bowel disease can be varied and protean. It is estimated that 1-2% of all patients with inflammatory bowel disease will develop episodes of acute pancreatitis during the course of the disease, with exocrine pancreatic insufficiency developing in 8-50% of cases [5]. Patients with inflammatory bowel disease, and those with ulcerative colitis in particular, are at increased risk of autoimmune pancreatitis. However, this association has been determined predominantly on the basis of retrospective studies [6, 18-21], which did not set out specifically to assess or determine the significance of serum IgG4 levels [22, 23].

In the sample described herein, 17.8% of patients had elevated serum IgG4 levels, a rate fourfold that reported by Takeuchi et al [24]. Only one patient in this study had IgG4-RD/autoimmune pancreatitis according to HISORt criteria [6], whereas Takeuchi et al did not report any such cases [24].

Elevated serum IgG4 levels in inflammatory bowel disease have been associated with active disease, treatment refractoriness, and presence of extraintestinal manifestations, such as primary sclerosing cholangitis [25]. Despite the small sample size, no association between elevated IgG4 levels and any of these events was found in the present study. In inflammatory bowel disease case series published thus far in the literature, disease activity has been defined predominantly on the basis of histopathological findings or by the presence of aggressive ulcerative colitis/Crohn’s disease requiring surgical intervention. High rates of extraintestinal manifestations such as primary sclerosing cholangitis were reported in these case series. There was no centripetal bias in the present sample, as all patients were recruited from a general outpatient inflammatory bowel disease clinic.

In this sample, elevated serum IgG4 levels were found in nine patients with ulcerative colitis, but in only one with Crohn’s disease. This between-group difference yielded a prevalence ratio of 16.42 (P = 0.0006). Virk et al [26] and Raina et al [27] reported similar results with analysis of tissue IgG4 levels, finding a greater prevalence of IgG4+ plasma cells among patients with ulcerative colitis, but neither study provided an in-depth analysis of serum IgG4 levels.

Diagnosis of IgG4-RD is based on clinical criteria (such as enlargement of affected organs), elevated serum IgG4 levels, and histopathological findings consistent with the disease (such as fibrosis, lymphoplasmacytic infiltration, and presence of ≥ 10 IgG4+ plasma cells in tissue samples) [8, 28]. In the absence of these criteria, as occurred in nine out of 10 patients in this sample, an alternative explanation for the isolated elevated serum IgG4 levels found in patients with ulcerative colitis would be a potential utility of this finding as a diagnostic biomarker, particularly in cases where the differential diagnosis between ulcerative colitis and Crohn’s disease is challenging. Further research is required to support this hypothesis [29].

Histological examination of colon biopsy specimens with immunohistochemical assessment was only available for two of the 10 patients with elevated serum IgG4 levels. Both specimens exhibited an IgG4+ plasma cell count below the cutoff value of 10 cells per high-power field.

Of the 26 tissue samples obtained, three displayed IgG4+ plasma cell infiltration, characterizing IgG4-positive colitis. However, serum IgG4 levels were normal in all three patients, which suggests dissociation between serum and tissue levels of IgG4. Park et al [7] identified two patients with IgG4+ plasma cells in colon tissue, both of whom had comorbid exocrine pancreatic insufficiency and only one of whom had serum IgG4 levels > 135 mg/dL. Ravi et al [6] reported four patients with autoimmune pancreatitis and inflammatory bowel disease, only one of whom had increased serum and tissue levels of IgG4. In the sample reported herein, three patients had IgG4-positive colitis, but normal serum IgG4 levels, and only one of these had active disease at the time of testing. Despite a paucity of published reports, a finding of elevated tissue IgG4 levels with no evidence of active disease is highly unusual [4, 27].

IgG4+ plasma cell infiltration of gastrointestinal tissue is associated with a Th2-type response [30]. This cell response pattern is most evident in the colon, which plays host to a diverse bacterial and parasitic resident microflora. Interaction between the intestinal microbiota and toll-like receptors in the dendritic cells of the bowel mucosa, with consequent activation thereof, plays a major role in the pathogenesis of ulcerative colitis [31, 32] and might explain the elevated serum IgG4 levels found in some patients in the present study.

In this sample, azathioprine therapy was significantly associated with elevated IgG4 levels (P = 0.0017), but not with disease activity (P = 0.8). In Brazil, azathioprine treatment of ulcerative colitis is indicated in corticosteroid-dependent patients or as an adjunct to biologic therapy. The fact that these patients would usually present with more active disease was not reflected in the disease activity scores used in this study, which were based on clinical activity (Crohn’s disease activity index and Lichtiger clinical-activity index) [19, 33]. The Lichtiger index has particularly high sensitivity for detection of clinical improvement in response to pharmacotherapeutic intervention [13]. Future studies should prioritize the use of endoscopic-based or combined (histological, endoscopic, and clinical) disease activity scores rather than clinical activity scores alone.

Ten (17.58%) of the 56 patients in this sample had exocrine pancreatic insufficiency, defined as a fecal elastase measurement of < 200 μg/g. This rate was similar to that reported in other studies [1, 2]. Exocrine pancreatic insufficiency did not correlate with disease activity, and therefore could not have been misdiagnosed as decompensated ulcerative colitis or Crohn’s disease. Oligosymptomatic exocrine pancreatic insufficiency is not unusual; exocrine pancreatic insufficiency may present without the classical clinical manifestations even in populations with chronic calcifying pancreatitis or diabetic patients, although this test has the greatest accuracy among the less invasive diagnostic modalities [34].

The mechanisms involved in the comorbid occurrence of inflammatory bowel disease and autoimmune pancreatitis have yet to be elucidated. It may constitute an extraintestinal manifestation of inflammatory bowel disease, a condition on the IgG4-RD spectrum (type 1 autoimmune pancreatitis), or even an extrapancreatic manifestation of type 2 autoimmune pancreatitis (pancreas-specific disease). No association between exocrine pancreatic insufficiency and elevated serum IgG4 levels was observed in this study. Although the small sample size is a concern, exocrine pancreatic insufficiency patients had normal pancreatic function. The etiology of exocrine pancreatic insufficiency was determined in two cases: convalescent stage of acute pancreatitis and obstructive pancreatitis secondary to intraductal papillary mucinous neoplasm. Case reports have implicated intraductal papillary mucinous neoplasm in the pathogenesis of IgG4-RD [35], a finding that was not replicated in this patient.

Limitations of this study include failure to conduct imaging studies (MRI, CT, or even endoscopic ultrasound) in all patients with exocrine pancreatic insufficiency and the prevalence study design, with no healthy control group and a small sample size. Thirty-one patients (approximately 55%) were already on corticosteroids, which may have induced a reduction in IgG4 levels. Multiple regression analysis found that the lack of previous use of corticosteroids was associated with elevated serum IgG4 levels (P = 0.047) [32]. Prospective studies are required to confirm or refute the hypotheses that elevated tissue IgG4 levels may denote a new phenotype of inflammatory bowel disease/ulcerative colitis (IgG4-related colitis) and that serum IgG4 measurements may serve as a biomarker for diagnosis of this novel subtype of colitis or of ulcerative colitis.

In conclusion, in patients with inflammatory bowel disease, and those with ulcerative colitis in particular, elevated serum IgG4 levels do not correlate with exocrine pancreatic insufficiency. There appears to be no association between disease activity and reduced fecal elastase or elevated serum IgG4, although the latter finding was more prevalent in patients with ulcerative colitis. This suggests a potential role for serum IgG4 elevation as a diagnostic biomarker for ulcerative colitis or even for a new phenotype of inflammatory bowel disease: IgG4-related colitis. Nevertheless, prospective studies are required to confirm or refute the hypotheses that elevated tissue IgG4 levels may denote this new phenotype of inflammatory bowel disease/ulcerative colitis.

## Author Notes

This article was based on the master's thesis of Dr. Ricardo Jacaranda Faria defended in 2013 in Universidade de Brasilia.
